# Supplementary material for: The Reaction of the Yeast Saccharomyces cerevisiae to Contamination of the Medium with Aflatoxins B2 and G1, Ochratoxin A and Zearalenone in Aerobic Cultures
Source: Int J Mol Sci. 2023 Nov 16;24(22):16401. doi: 10.3390/ijms242216401 (PMC10671187; doi:10.3390/ijms242216401)

Supplementary Materials:

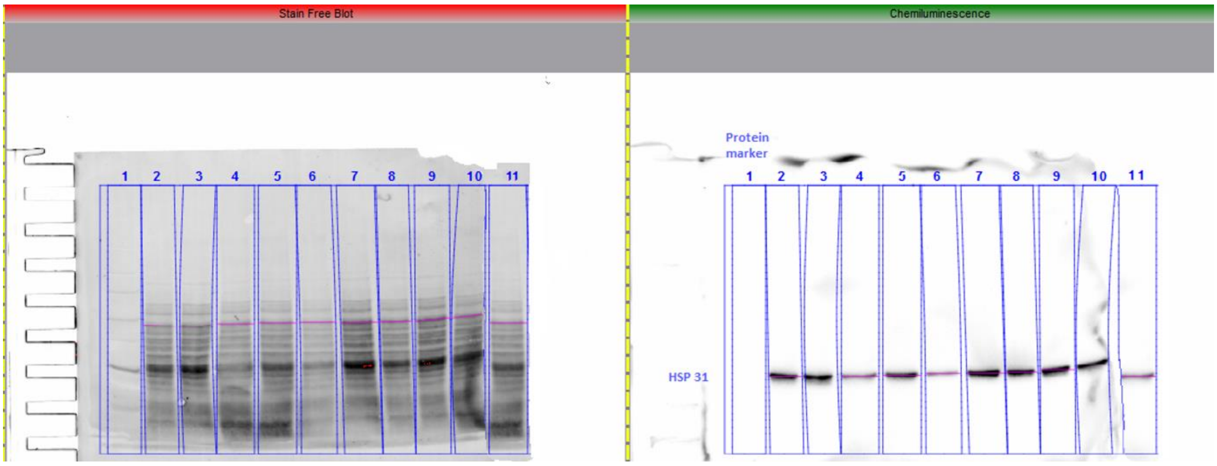

Figure S1: The effect of lower doses of mycotoxins on the production of HSP31 (10µl per well) in aerobic conditions. Image based on graphical analysis performed with Image Lab 6.1 Software comparing stain free blot with the image after chemiluminescence detection of proteins. Lane 1 – protein size marker, lane 2- control, lanes 3, 4, 5 – AFB_2 + AFG_1, lanes 6, 7, 8 – OTA, lanes 9, 10, 11 – ZEA.

This figure was created based on the two below original figures of Western Blotting:

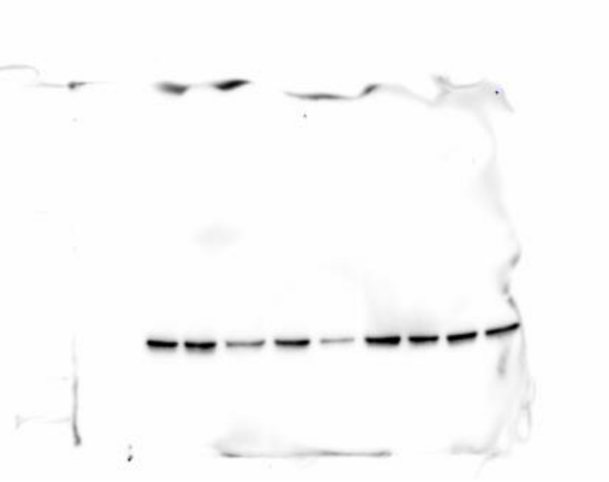

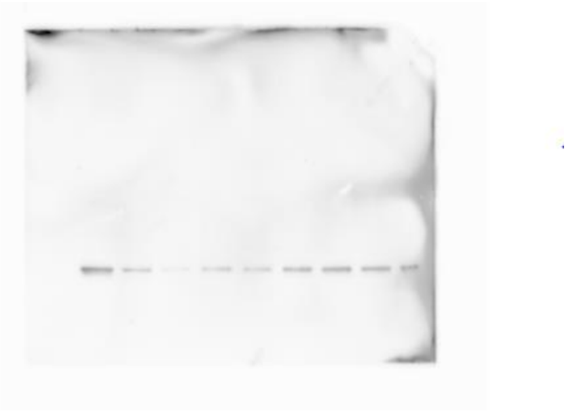

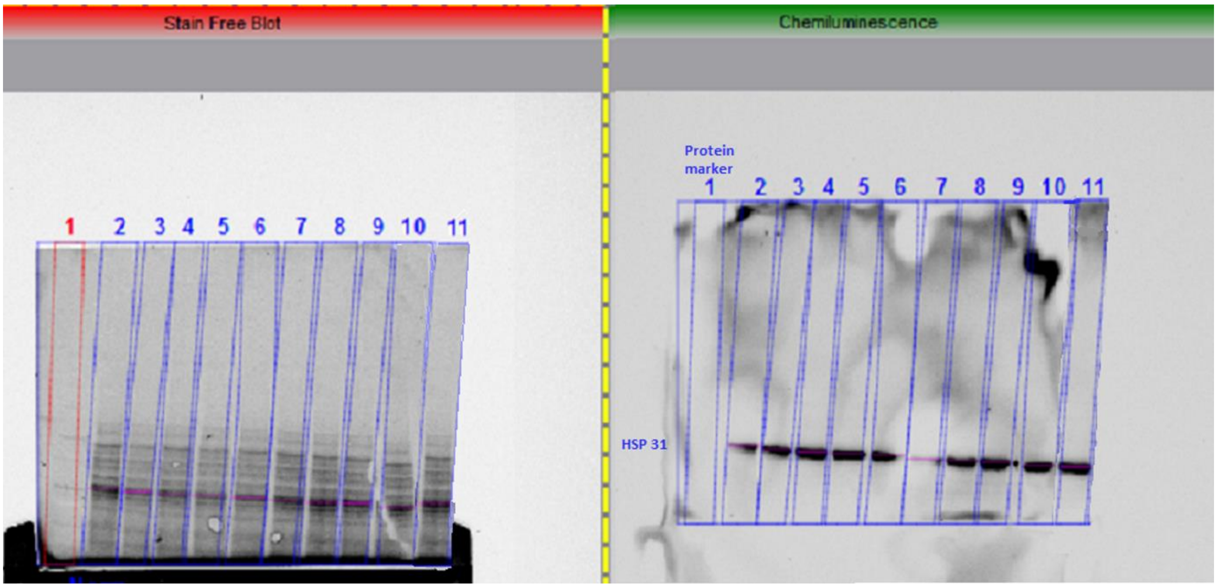

Figure S2: The effect of higher doses of mycotoxins on the production of HSP31 (10µl per well) in aerobic conditions. Image based on graphical analysis performed with Image Lab 6.1 Software comparing stain free blot with the image after chemiluminescence detection of proteins . Lane 1 – protein size marker, lane 2- control, lanes 3, 4, 5 – AFB_2 + AFG_1, lanes 6, 7, 8 – OTA, lanes 9, 10, 11 – ZEA.

This figure was created based on the two below original figures of Western Blotting:

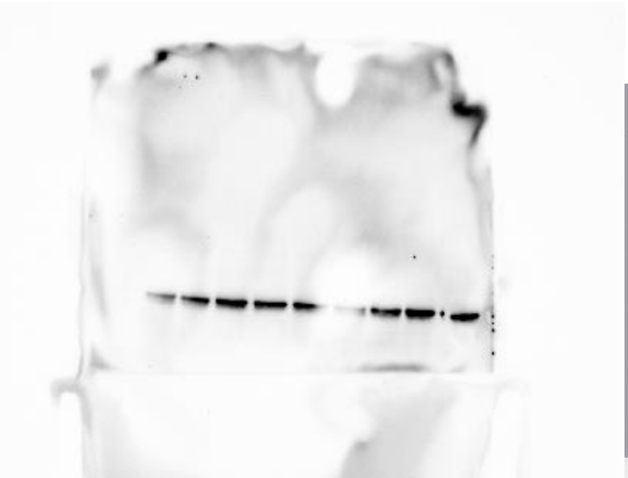

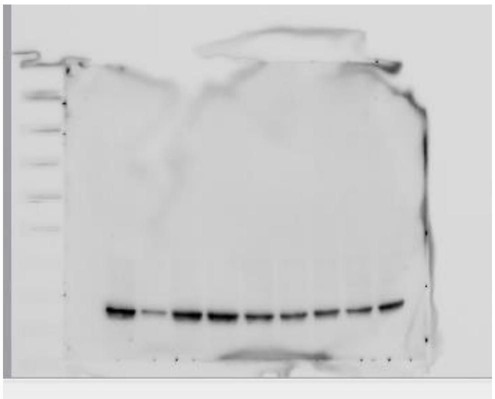

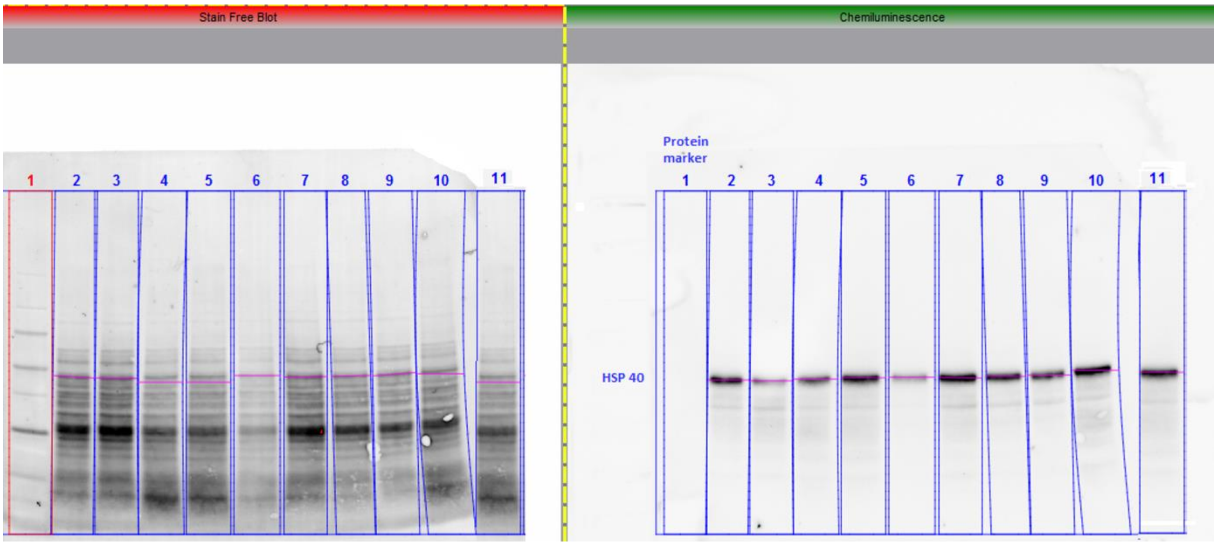

Figure S3: The effect of lower doses of mycotoxins on the production of HSP40 (10µl per well) in aerobic conditions. Image based on graphical analysis performed with Image Lab 6.1 Software comparing stain free blot with the image after chemiluminescence detection of proteins. Lane 1 – protein size marker, lane 2- control, lanes 3, 4, 5 – AFB_2 + AFG_1, lanes 6, 7, 8 – OTA, lanes 9, 10, 11 – ZEA.

This figure was created based on the two below original figures of Western Blotting:

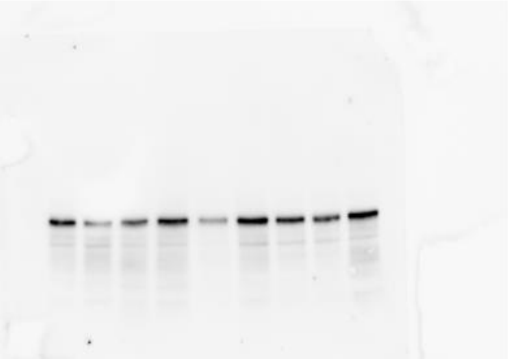

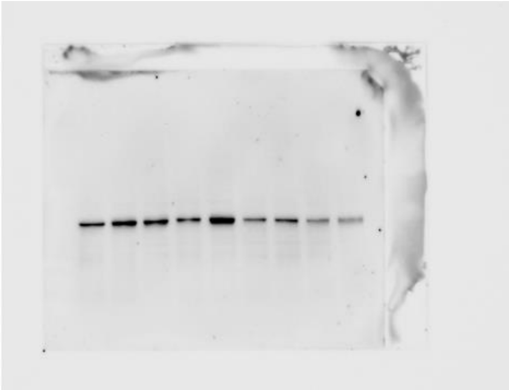

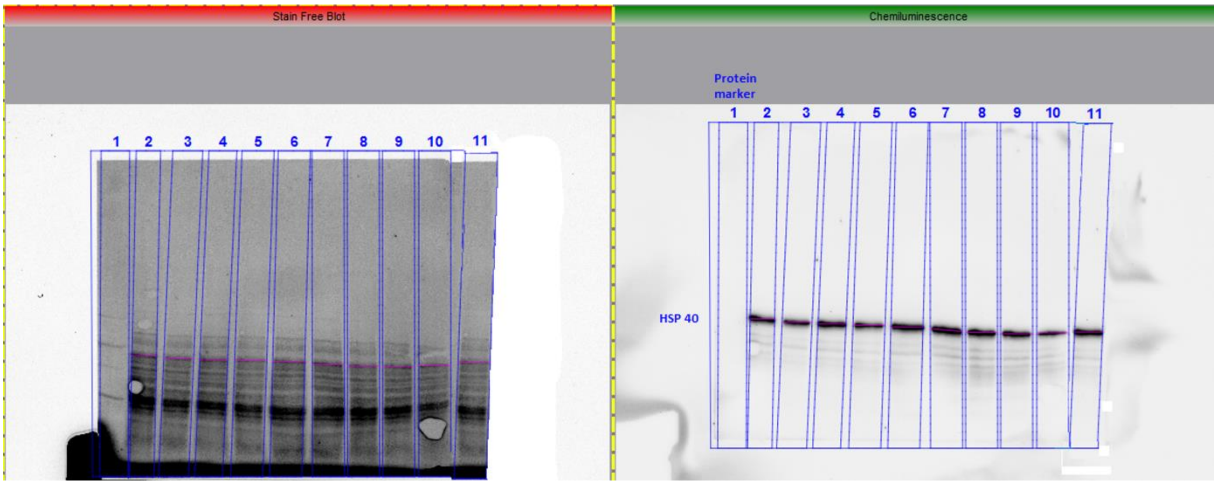

Figure S4: The effect of higher doses of mycotoxins on the production of HSP40 (10µl per well) in aerobic conditions. Image based on graphical analysis performed with Image Lab 6.1 Software comparing stain free blot with the image after chemiluminescence detection of proteins. Lane 1 – protein size marker, lane 2- control, lanes 3, 4, 5 – AFB$_2$ + AFG$_1$, lanes 6, 7, 8 – OTA, lanes 9, 10, 11 – ZEA.

This figure was created based on the two below original figures of Western Blotting:

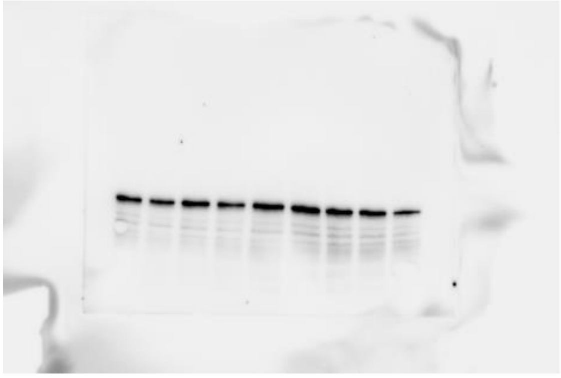

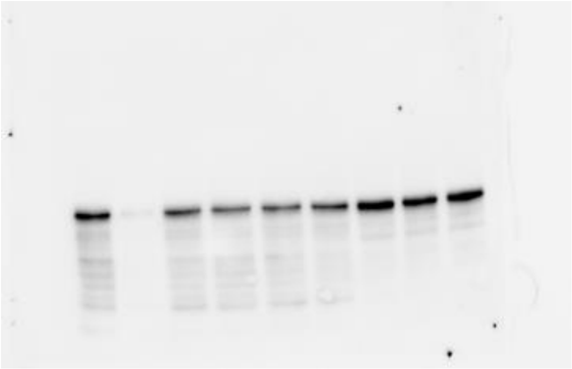

Supplement: Supplementary file 1 [file ijms-24-16401-s001.zip › ijms-2671165-supplementary.pdf]
